# The Effect of School Psychologists and Social Workers on School Achievement and Failure: A National Multilevel Study in Chile

**DOI:** 10.3389/fpsyg.2021.639089

**Published:** 2021-02-23

**Authors:** Verónica López, Karen Cárdenas, Luis González

**Affiliations:** ^1^School of Psychology, Pontificia Universidad Católica de Valparaíso, Valparaíso, Chile; ^2^Center for Research in Inclusive Education, Viña del Mar, Chile

**Keywords:** school psychologists, social workers, school achievement, school failure, multilevel, Chile

## Abstract

School achievement and failure have become growing political and social concerns due to the negative consequences of school failure for individuals and society. The inclusive educational movement, which calls for equal access, permanence, participation, and promotion of all students worldwide, poses many challenges for schools and school systems. As a public policy strategy, some countries have provided additional funds for incorporating non-teaching professionals such as school psychologists and social workers in regular K-12 schools. However, there is lack of research on the effects of these psychosocial professionals on student outcomes. This national multilevel study explored the effect of psychologists (*n* = 8,469) and social workers (*n* = 3,524) on indicators of eighth-grade (*n* = 147,531) and 10th-grade (*n* = 106,347) students' academic achievement and dropout in Chile. A multilevel secondary analysis was performed using national records of non-teaching professionals working as school staff members, achievement scores on the national SIMCE test, and dropout rates based on official records. Results showed that after controlling for individual and school variables known to affect achievement and dropout, schools with psychologists and social workers working as staff members had lower short- and long-term dropout rates. The presence and higher number of school psychologists per school was positively associated with higher math achievement, with a reduced effect in low-SES schools. Lower-SES schools with more social workers had higher math scores. These results support policies that increase funding for school psychologists and social workers, since their incorporation partly explains better school achievement and less school failure when controlling for individual and school characteristics, but emphasize the need to further explore the mechanisms through which school achievement and failure are developed with the support of psychologists and social workers in schools. We discuss the need to regulate the type of prevention and intervention strategies from a whole-school, evidence-based approach, as well as to incorporate psychosocial training modules and comprehensive guidelines as part of professional training programs and as certified requisites for working in schools.

## Introduction

Access to education, permanence, participation, promotion, and graduation are fundamental pillars for the achievement of an inclusive education (Ascorra and López, 2019). According to UNESCO UNICEF (2007), every girl or boy has the right to go to school, access relevant learning, and be treated with dignity under conditions of equality. School systems around the world must guarantee that their students remain in the school system and provide engaging learning opportunities for all students (Ainscow, 2019).

During the 1990s, concern about integrating quality measurements in education began to rise internationally, based on the need to provide quality learning experiences for all students to reduce inequities (Liu et al., 2019). These discussions were strongly reinforced by international organizations, which urged their member countries to develop mechanisms for the evaluation, monitoring, and improvement of education from an economic perspective and rationality, wherein the main objective was to reduce poverty (Anaya, 2019; Prieto, 2019). Initially, this conception of quality considered only academic performance, but it later began to incorporate other dimensions of learning from a more holistic perspective (Cohen and Espelage, 2020).

Historically, academic performance or achievement has been considered one of the most important indicators in terms of quality and equity in education, because it has been shown to be a predictor of the quality of life of students (Organisation for Economic Cooperation Development., 2016; Liu et al., 2019). Traditionally, the international literature understood academic achievement as learning outcomes measured through a standardized assessment. These assessments can be at the school level, considering national or district assessments, or at the student level, considering their individual performance (Ruiz et al., 2018; Granvik et al., 2020). Although initially academic performance or achievement was considered the sole indicator that could verify the achievement of learning, Coleman et al. (1966) report, which verified the relevance of the socioeconomic status (SES) of families to student performance, paved the path for including other relevant factors inside and outside the school.

During the past 30 years, numerous studies have been carried out regarding the factors that affect academic performance or achievement. These studies have made it possible to elucidate factors internal to the school that can influence this phenomenon, even acting as moderators of SES (Liu et al., 2018, 2019; Granvik et al., 2020). Some factors shown to have an effect on intraschool performance or achievement are school leadership, high teacher expectations, teacher–student support (Thapa et al., 2013; Granvik et al., 2020), student and parent participation (Boonk et al., 2018; Lei et al., 2018), school climate (Astor et al., 2009; Thapa et al., 2013; Ruiz et al., 2018), and specialized programs or interventions (O'Connor, 2018; Arslan and Coşkun, 2020; Mulhern, 2020).

With time and the persistent and progressive movement toward inclusion and equity in education, the evaluation of educational quality outcomes began considering school failure and factors that act as barriers for students' promotion and permanence in the regular school system (Ainscow, 2019). School dropout is considered the utmost indicator of school failure, but it is understood as a culminating milestone of a progressive process of distancing from the school (Ministerio de Educación, 2020a). External and internal aspects of the school intervene in this process, which constitute mechanisms of exclusion (Román, 2013; Robison et al., 2017; Gubbels et al., 2019), in the sense that the students don't drop out, but instead are pushed out due to internal school factors or pulled out due to external community and social factors (Doll et al., 2013). The intraschool variables are factors such as school belonging, social and pedagogical support from teachers, and availability of institutional support, among others. On the other hand, among extraschool variables are the socioeconomic level of the families of origin, parents' educational level, and place of residence, among other aspects (Román, 2013; Pate et al., 2016; Robison et al., 2017). This is how school dropout is constituted as a complex and multicausal phenomenon (Román, 2013; Archambault et al., 2017; Hernández and Diaz, 2017; Gubbels et al., 2019). Research on school dropout has shown risk factors that can propitiate its outcome (Gubbels et al., 2019). In this regard, factors external to school, such as family problems, the need to work, living in vulnerable neighborhoods, physical or mental health problems, or criminal problems, are some factors that affect dropout (Iachini et al., 2016; Gubbels et al., 2019; Parviainen et al., 2020). Likewise, factors internal to the school such as absenteeism, repetition, low academic performance, expulsion or suspension policies, negative school climate or little socioemotional support are some incident factors (Pate et al., 2016; Tello and Lonn, 2017; Filippello et al., 2019; Gubbels et al., 2019).

However, the phenomenon of school dropout does not occur in the same way in all groups of students. Research has shown that the groups with the highest risk of dropping out are those with low socioeconomic SES, ethnic groups, and immigrants (Duncan and Murnane, 2011; Román, 2013; Archambault et al., 2017; Hernández and Diaz, 2017; Robison et al., 2017; Tello and Lonn, 2017; Ministerio de Educación, 2020a). This poses challenges for schools and public policy makers, who might have difficulty incorporating these both pedagogically and socioculturally (Archambault et al., 2017; Robison et al., 2017).

In this regard, schools are challenged to meet the demands of the inclusive educational movement, which calls for equal access, permanence, participation, and promotion of all students worldwide, poses many challenges for schools and school systems. One of these challenges is how to identify and attend to the psychosocial needs of possible risk groups according to their internal and external characteristics (Pate et al., 2016; Archambault et al., 2017; Tello and Lonn, 2017), and how to best serve them through adequate prevention and intervention strategies. As a public policy strategy, some countries have provided additional funds for incorporating non-teaching professionals such as school psychologists and social workers in regular K-12 schools. The rationale is that incorporating these support professionals in regular school systems can help identify and intervene in these phenomena, and through this, influence quality indicators of school achievement and school failure, such as academic performance and school dropout (Hernández and Diaz, 2017; Tello and Lonn, 2017; Kuperminc et al., 2019).

Organisation for Economic Cooperation Development. (2019), the average school enrollment rate of its member countries in the population between 15 and 18 years old is 84.5%. Specifically, in Chile, this figure corresponds to 80.9%, which is below the OECD average but higher than the reality of other Latin American countries such as Mexico or Brazil. On the other hand, the dropout figures in Chile for 2010 reached <1%, being one of the lowest figures in Latin America (UNESCO., 2013). However, the country is recognized for having one of the most segregated education systems in the world (Organisation for Economic Cooperation Development., 2016). This is how, according to a report prepared by the Study Center of the Ministry of Education, it is observed that the highest rate of school dropout occurs mainly in quintiles I and II; that is, the lowest-income quintiles in the country (Ministerio de Educación, 2020a).

This scenario of deep segmentation has emphasized the need to develop compensatory policies that from a focused perspective seek to cushion the learning gaps generated by the system (Almonacid, 2000; Bellei, 2007; García-Huidobro, 2007; Jorrat and Rojas, 2016). In this context, since the beginning of this century, policies such as the preferential school voucher law (known as SEP, an acronym for Ley de Subvención Escolar Preferencial, Law Nr. 20.248., 2008) or the pro-retention law (Law Nr. 19.873., 2003) were created. These policies assume that low-SES students (identified in the Chilean policies as “vulnerable students”) are more expensive to educate, and therefore, schools with more low-SES students need a bigger budget. In Chile, this additional funding, given the market-based voucher financing of the school system (not per school but per student enrolled and attending schools), is delivered via a double or triple voucher per student identified as socioeconomically vulnerable. These funds, provided mainly from the SEP law, have allowed schools to acquire educational materials and hire new professional support, which historically were not in the school system.

Prior to this period, some public initiatives in education, such as the Schools and High Schools for All program, gradually made it possible to incorporate psychologists and social workers into education. Timidly, other similar programs counted on their presence in a counseling mode, such as the Liceos Prioritarios program. Since the end of the 90s with differential groups, and later with the School Integration Programs (PIE) aimed at students with disabilities, psychologists have been approaching the school space in their professional practice. However, it was not until the SEP law that the schools, along with the hiring of teaching staff for pedagogical support, could independently hire psychosocial support professionals. Many schools jointly hired psychologists and social workers to train what came to be called “psychosocial pairs” (López and Carrasco, 2018).

Since 2010, when the SEP law began implementation, psychosocial pairs have grown exponentially in publicly funded schools (Raczynski et al., 2013; López et al., 2020). Between 2010 and 2018, there was an increase of more than 800% in psychosocial professionals hired in schools. According to official records, the 1,704 professionals hired in 2010 grew to more than 14,000 professionals by 2018 (López et al., 2020).

Although the working conditions of these professionals have improved, many of them have 10-month contracts (March to December, summer holidays not included) that are renewed annually, which generates job instability that discourages the permanence of these professionals (López and Carrasco, 2018). López et al. (2020), when carrying out an analysis regarding the socio-labor characteristics of these professionals, found that in 2018, around 45% of psychologists and social workers had a professional practice experience of 0 and 4 years, probably due to working conditions. This means that today, although there are psychosocial professionals in 60% of the schools that receive funding from the state (public and subsidized private), these tend to be recently graduated young professionals whose first work exercise is to work in schools, which was evidenced by López et al. (2020), who found professionals who had stayed < 2 years in the same establishment grew from 0% in 2015 to 49% in 2018.

Moreover, the progressive incorporation of psychosocial professionals in Chilean schools occurred without an explicit intervention model, but under the assumption of support directed at individual students. On the one hand, the SEP law, by focusing on the provision of additional resources to schools, did not and still does not incorporate a psychosocial intervention model (Law Nr. 20.248., 2008), although it does emphasize the need for “psychosocial supports” but with undefined intervention models and plans. Rather, this Law provides a model of school management in four areas –one of them is school climate- but, following a decentralized logic of education, does not mandate nor suggest any given program or intervention model. It was not until 2017 that the Ministry of Education provided guidelines for the formation of school climate teams (Ministerio de Educación, 2017). In this document, some of the actions to be carried out by the psychosocial pairs were made explicit by way of suggestions and orientations, but maintained a logic of individual diagnosis and intervention. On the other hand, the professionals hired by PIE funds present a similar situation. The regulations for PIE are very clear as to which disability diagnoses (special educational needs or SEN) are eligible for funding, and who and how must perform the diagnoses (Decree No. 170, 2010). However, it was not until 2015 that guidelines were established regarding support plans for these students, which emphasized the formation of “classroom teams” with co-teaching tasks (Decree No. 83, 2015). Despite this, the student support functions of the school psychologists hired under PIE funding are still not delimited. Guidelines elaborated in 2016 were still not clear as to what a support plan for PIE psychologists should look like, but only mentioned collaborative actions with classroom teachers (Ministerio de Educación, 2016).

In this context, the national policy, instead of proposing an intervention model with an evidence-based comprehensive school approach (Dimmit and Robillard, 2014), assumes that psychologists and social workers should work individually with students with the greatest needs. In most schools, the school administrators and school principals in practice define the type of work, roles, and functions of these professionals, and these definitions are frequently based on deficit theories and individual approaches to intervention (López and Carrasco, 2018). Thus, the predominant intervention logic has been intervention in the “case” identified or reported by the school and generally, without the characteristic of a “pair” or “team,” but rather through specific professional interventions of each professional directly with specific students, who are treated as and even called “cases” (López and Carrasco, 2018). In this way, a pathologizing approach has been instituted, wherein students who do not respond adequately to the official curriculum are diagnosed, intervened with, or referred (Sandoval and Lamas, 2017; Cárcamo-Vásquez et al., 2020), just as if they were in a medical system.

Indeed, a national study by Center for Research in Inclusive Education (Ascorra et al., 2019) recently showed that most schools, regardless of their dependence, predominantly carry out actions of “putting out fires” or paying individual attention to students, to the detriment of group intervention actions, coordination with other professionals, or networking. These types of intervention focused on individual interventions correspond to level 3 of tiered whole-school approaches (Dimmit and Robillard, 2014), which are recommended only for 5–10% of a school's student population. Prior to these actions, tiered whole-school approaches recommend tier 1 actions, which are primary prevention actions aimed at all students and the school community that promote the well-being of students and the school, both curricularly and psychosocially, articulating both dimensions systematically. In the case of students who for various reasons do not respond well to level 1 actions, it is recommended to move to level 2 actions, or secondary prevention, which generally correspond to group intervention actions on groups of students identified as at risk or with greater socioemotional needs (Dimmit and Robillard, 2014). In the case of Chile, the evidence shows that the pyramid in whole-school approach models is inverted, because Chilean psychosocial professionals most frequently implement tier 3 interventions.

However, the entry of these professionals is an opportunity for schools to attend to the various problems and needs of students and incorporate contextual variables into the school (Gatica, 2016). Research has shown that psychosocial pairs have been able to address different issues associated with vulnerability, enabling access to state benefits, access to health networks, attention to psychosocial needs, and protection of the rights of girls, boys, and young people, among other aspects (López et al., 2011a,b; Cádiz and Manríquez, 2015; Gatica, 2016; Jorrat and Rojas, 2016; López and Carrasco, 2018; Cárcamo-Vásquez et al., 2020).

However, in the context of a mode of intervention that focuses on tier 3 interventions and is not based on evidence-based approaches, it is necessary to understand the consequences or effects of progressive incorporation of psychosocial professionals on schools in terms of improvement in some key dimensions of educational quality such as permanence and school achievement.

The international literature shows few studies that evaluate or expose the effects of the incorporation of other support professionals in schools such as psychologists, social workers, or school counselors on school achievement and failure (Mulhern, 2020). Studies related to this issue have instead focused on verifying the impact of specific programs in some schools or districts. These programs have generally been developed from a counseling model, with the participation of school counselors and teachers, and with a lower percentage of psychologists and social workers (O'Connor, 2018; Healy et al., 2020).

The implementation of intervention or counseling programs has been shown to have similar characteristics. Most of these programs have focused on the development of skills or socioemotional competencies, and there are also—to a lesser extent—others that aimed to improve academic indicators or other aspects (Ballard et al., 2014; Franklin et al., 2017; Healy et al., 2020). Although these programs were implemented from a promotional approach, they might also include large- or small-group tier 2 interventions. Programs that have been developed exclusively from an individual approach have been shown to have less impact on students (Franklin et al., 2017; Healy et al., 2020). Likewise, these programs, despite having effects on different groups of students, have been shown to have a higher effect on higher-risk students, who generally present a gap in achievement indicators compared to their peers (Hoagwood et al., 2007; Franklin et al., 2017; Mason and Dye, 2017; O'Connor, 2018; Healy et al., 2020).

The effects of programs linked to psychosocial supports have been varied. Some studies have shown a decrease in behaviors of physical and sexual violence at school (Kernsmith and Hernandez-Jozefowicz, 2011; Healy et al., 2020), discrimination against minorities (Cohen et al., 2006; Tello and Lonn, 2017; Mulhern, 2020), and risk behaviors or mental health problems (Baskin et al., 2010; Ballard et al., 2014; Franklin et al., 2017; O'Connor, 2018), alongside an increase in prosocial behaviors (Kuperminc et al., 2019). Along the same lines, some studies have shown an increase in school engagement and a positive assessment of relationships at school (Kuperminc et al., 2019; Healy et al., 2020; Mulhern, 2020). Other studies found improvements in the subjective well-being of school members, which has improved students' academic performance (López et al., 2017; Arslan and Coşkun, 2020).

Regarding the effect of non-teaching personnel on indicators of school achievement and school failure, Mulhern (2020) found that the presence of school counselors had a negative effect on school suspensions, reducing the probability that students who participated in counseling would be suspended. Likewise, a positive effect on the probability of accessing, remaining in, and graduating from university education was evidenced. This has been supported in previous studies (Poynton and Lapan, 2017). The effects were shown to be greater in students with lower SES or school performance. Other programs have shown a reduction in the likelihood of school dropout (Harris and Franklin, 2003) and number of school suspensions (Ballard et al., 2014).

On the other hand, specialized programs in student groups have been shown to have an impact on academic performance. These programs have shown that adaptation to the local context of the school and positive appreciation of the program by students are essential for their success (Baskin et al., 2010; Yeager and Walton, 2011; Henry et al., 2017; Mason and Dye, 2017). In this way, the literature shows that it is important for schools to have defined intervention models and programs to improve school experience and performance.

In this context, the purpose of this study was to analyze the effect of the presence of school psychosocial professionals on indicators of school achievement and school failure, beyond students' individual and school characteristics known to affect achievement and dropout.

## Materials and Methods

### Participants

The dataset of the Chilean national assessment system (Sistema de Medición de la Calidad de Educación, known as SIMCE) in its 2017 version was used. SIMCE is a standardized testing system that provides nationwide information about students and schools that participate in the regular educational system. In this study, participants were eighth- and 10th-grade students and their parents. For this study, the students' tests scores and questionnaire were used; the latter provided self-report information related to their school experiences. Additionally, the parents' questionnaire provided information that allowed a sociodemographic characterization of the students and their families. Of the initial student sample, 81% had matched information with the parents' questionnaire. Additionally, the 2017 National Record of Educational Assistants was used. This data allowed identification of the psychosocial professionals hired by all Chilean schools, by type of professional, type of funding (SEP or PIE), and number of professionals per school. The process of merging both datasets produced a loss of 3% of the matched sample. Finally, the 2018 and 2019 General Information System of Students database was used. This dataset provided information about students' likelihood of dropping out during the next 2 years of schooling, by providing information regarding if a student was studying in the school system in the following 2 years.

Finally, we restricted our study to students who had no missing values in the study variables. The final sample consisted of 147,531 eighth-grade students and 106,347 10th-grade students from public, subsidized private, and private schools in Chile, representing 70% of the initial sample, along with 8,469 psychologists and 3,524 social workers working in 2017 as non-teaching school staff members in 5,091 of the 6,358 public, subsidized, and private schools in the total sample. To characterize the sample, descriptive statistics are reported for students and psychosocial professionals in Tables 1, 2, respectively.

**Table 1:** Means and standard deviation of individual-level study variables by full sample and grade.

**Variable**	**Total sample**		**8th grade**		**10th grade**		**Differences (8th−10th)**
	**Mean (or %)**	**SD**	**Mean (or %)**	**SD**	**Mean (or %)**	**SD**	
Math SIMCE score	268.72	55.52	262.11	48.72	277.91	62.65	−15.80^***^
Language SIMCE score	252.16	50.45	246.69	49.30	259.78	51.04	−13.10^***^
Short–term dropout	0.02	0.14	0.01	0.11	0.03	0.16	−0.01^***^
Long–term dropout	0.02	0.13	0.01	0.11	0.03	0.16	−0.01^***^
Female	0.52	0.50	0.50	0.50	0.53	0.50	−0.03^***^
Age	14.16	1.17	13.32	0.62	15.33	0.61	−2.01^***^
Individual SES	0.06	0.87	0.02	0.86	0.12	0.88	−0.10^***^
Indigenous ascendance	0.13	0.34	0.14	0.35	0.12	0.33	0.02^***^
Attendance	93.59	5.26	93.84	5.14	93.24	5.39	0.60^***^
School Motivation	0.03	0.67	0.03	0.66	0.03	0.68	−0.00

*Standard errors in parentheses. T–tests performed for the estimation of differences by grade*.

*** *p < 0.001*.

**Table 2:** Means and standard deviation of descriptive variables of psychologists (*N* = 8,469) and social workers (*N* = 3,524).

	**Psychologists**		**Social workers**		**Total**	
**Variable**	**Mean (or %)**	**SD**	**Mean (or %)**	**SD**	**Mean (or %)**	**SD**
Female	75.66	–	86.27	–	78.72	–
Age	34.29	7.29	34.82	7.86	34.97	7.46
Years of experience	3.92	4.59	3.59	4.28	3.83	4.51
Type of contract						
Indefinite contract	35.57	–	34.41	–	35.23	–
Fixed term	62.65	–	63.24	–	62.82	–
Fee contract	1.78	–	2.35	–	1.94	–
Working hours per week	30.63	13.45	30.73	14.03	30.66	13.62

### Measures

#### Dependent Variables

##### Math and Language SIMCE Test Scores

Scores of the standardized 2017 SIMCE test of mathematics and language for eighth- and 10th-grade students. Since 1999, the SIMCE scores are scaled based on Item Response Theory, with a national standardized mean of 250 points and a standard deviation of 50, using 1999 as the baseline year.

##### Dropout Status

Two indexes were created for students' dropout status. First, ***short-term dropout*** was computed as a dichotomous variable took the value of 1 if a student who was enrolled in a school in 2017 left school before finalizing the school year and took the value of 0 if the student finished the school year. Second, a ***long-term dropout*** was computed as a dichotomous variable that took the value of 1 if a student who was enrolled in a school in 2017 was not enrolled in any school in 2018 and 2019, and took the value of 0 if the student was enrolled in any school in Chile in 2018 or 2019.

#### Independent Variables: Individual Characteristics

##### Gender and Age

The students' gender and age were obtained from the official records of schools.

##### Socioeconomic Status

SES was obtained through the parents' questionnaires of the 2017 SIMCE test, wherein parents responded about their family income and the education level of the student's father and mother. A standardized index was computed.

##### Indigenous Ascendance

Each student's mother responded if she identified with any of the ethnic minorities recognized by law in Chile. This variable was codified as a dichotomous index: 1 indicates the student's mother belongs to any ethnic minority, and 0 indicates otherwise.

##### Attendance

Students' school attendance was obtained from the official school records.

##### School Motivation

An index of school motivation was constructed using seven items from the students' questionnaires with a 4-point Likert scale regarding their agreement from 1 (*strongly disagree*) to 4 (*strongly agree*) of the 2017 SIMCE database. The items were “I make an effort to do well in all subjects”; “I have fun learning new things in class”; “I make an effort to understand what is taught in class”; “I make an effort to have good grades”; “I like to study”; “I always do my homework”; and “Learning what is taught in class is very important for me.” The confirmatory factor analysis showed a good fit of the model: χ^2^(12) = 13,740.75, *p* < 0.001, RMSEA = 0.068, CFI = 0.975, TLI = 0.956.

#### Independent Variables: School Characteristics

##### Percentage of Female Students

The proportion of female students was computed for each school.

##### School Socioeconomic Status

The school SES was obtained from the 2017 SIMCE database, which classifies each school according to a school vulnerability index and the family income and schooling years of the enrolled students' parents. The school SES index has five categories (1 = *low SES*, 2 = *mid-low SES*, 3 = *mid SES*, 4 = *mid-high SES*, 5 = *high SES*).

##### Percentage of Vulnerable Students

This index was computed as the proportion of a school's enrolled students in 2017 who had an individual SES index that was one standard deviation below the school's mean.

##### Attendance (School Average)

The school rate of attendance was obtained by calculating the average of the enrolled students' attendance.

##### School Funding

The schools were classified according to their funding. This index has three categories (1 = *public*, 2 = *subsidized private*, 3 = *private*).

##### Rural School

A dichotomous index was codified to classify the schools. This variable takes the value of 1 if the school was in a rural zone and 0 if it was in an urban zone.

##### Type of School According to Hiring Policy

The schools were classified in four categories according to their policy of hiring psychosocial professionals. The first category was “no psychosocial professionals” if the school did not have any psychologist or social worker hired in 2017. The second category was “psychologist only” if the school hired at least one psychologist but had no social workers hired in 2017. The third category was “social worker only” if the school hired at least one social worker but had no psychologist hired in 2017. The fourth category was “psychosocial pairs” if the school hired at least one psychologist and one social worker in 2017.

##### Number of Psychosocial Professionals Hired

The total number of psychologists and social workers hired in each school was obtained from the 2017 National Record of Education Assistants.

##### Percentage of Psychosocial Professionals Hired With Indefinite Contract

The percentage of psychosocial professional hired with indefinite contracts was obtained from the 2017 National Record of Education Assistants.

##### Percentage of Psychosocial Professionals Hired With Fixed-Term Contract

The percentage of psychosocial professional hired with fixed-term contracts was obtained from the 2017 National Record of Education Assistants.

##### Percentage of Psychosocial Professionals Hired With SEP Funds

The percentage of psychosocial professional hired with SEP funds was obtained from the 2017 National Record of Education Assistants.

##### Percentage of Psychosocial Professionals Hired With PIE Funds

The percentage of psychosocial professional hired with PIE funds was obtained from the 2017 National Record of Education Assistants.

### Analytic Plan

Statistical analyses were carried out using Stata 13. The syntax and statistical processing can be found at https://mfr.osf.io/render?url=https%3A%2F%2Fosf.io%2Fnwp5r%2Fdownload. First, we viewed the descriptive data on each variable. Later, we performed a two-level linear multilevel analysis using math and language scores as dependent variables. The predictor variables took into consideration individual factors (level 1) and school factors (level 2). At the student level, we included the sociodemographic characteristics. At the school level, the variables were obtained by averaging the reported individual-level variables and included the schools' urbanicity and type of funding, as well as the key variables of the study related to the psychosocial professionals. Additionally, we performed a quantile regression analysis on the 25th, 50th, 75th, and 90th quantile of test achievement, to test if there were differentiated effects of the presence of psychosocial professionals on the mentioned performance groups. Finally, we performed a two-level logistic multilevel analysis using the dropout index as the dependent variable. To test the contribution of psychosocial professionals to academic performance and dropout, we estimated four models, considering their presence (Model 1), the number of school psychologists and social workers in schools (Model 2), their type of contract (Model 3), and their source of funding (Model 4).

### Ethical Considerations

This study was approved by the institutional review board of the first author's institution. Deidentified information from all datasets preserved the confidentiality of the students. We used an obfuscated identifier to merge the databases.

## Results

### Descriptive Statistics of Study Variables

Table 1 shows the means and standard deviations of individual-level variables for the total sample and by grade level. The mean tests scores of the eighth and tenth grade sample were about 10 to 20 points above the national mean of 250, except for the language tests of eighth grade students, which were almost 4 points below. The sample of 10th-grade students had higher short- and long-term dropout rates, a higher proportion of female students, higher SES, a lower proportion of students with Indigenous backgrounds, and a lower attendance rate. There were no statistically significant differences between grades in the school motivation index.

Descriptive statistics related to the relevant school-level study variables are reported in Table 3, in which we estimated the differences among schools by their school SES. With respect to the type of schools according to their hiring policy, schools with high and mid-high SES were more likely to have no psychosocial professionals hired or only psychologists. On the other hand, low and mid-low SES schools had a greater proportion of psychosocial pairs hired. There were no differences in the proportion of schools with only social workers. When considering the mean number of psychologists hired by school SES, mid-low SES schools had the highest mean and mid-high SES schools had the lowest mean, with no significant differences among the other categories. High SES schools had the highest mean proportion of professionals with indefinite contracts, and low and mid-low SES schools had the highest mean proportion with fixed-term contracts. Mid-low and mid-SES schools had the highest mean proportion of psychosocial professionals hired with SEP funds. Finally, there was a lower proportion of professionals hired with PIE funds in schools with higher SES.

**Table 3:** Percentages, means (and standard deviations) of school–level study variables for the whole sample and by school–SES.

	**Whole sample**	**School SES**					
		**Low (1)**	**Mid–low (2)**	**Mid (3)**	**Mid–high (4)**	**High (5)**	***Post–hoc***
**Variables**		**(*N =* 1,545)**	**(*N =* 2,078)**	**(*N =* 1,430)**	**(*N =* 645)**	**(*N =* 265)**	
Type of schools							
No psychosocial professionals hired (%)	19.1	16.1	11.9	21.8	33.3	44.5	5>4>3>1>2
Only psychologists (%)	36.6	30.3	30.8	41.6	52.7	52.5	5, 4>3>2, 1
Only social workers (%)	1.9	2.0	2.2	2.0	1.4	0.8	1=2=3=4=5
Psychosocial pairs (%)	42.4	51.7	55.2	34.7	12.6	2.3	1, 2>3>4>5
Number of psychologists hired	1.4 (1.1)	1.2 (0.9)	1.5 (1.2)	1.4 (1.2)	1.1 (1.1)	1.2 (1.7)	2>1, 3, 5>4
Number of social workers hired	0.6 (0.7)	0.7 (0.7)	0.7 (0.8)	0.4 (0.7)	0.2 (0.4)	0.0 (0.2)	1, 2>3>4, 5
Percentage of psychosocial professionals with indefinite contract	28.6 (37.5)	23.7 (34.7)	29.0 (35.9)	30.7 (35.4)	30.7 (41.8)	38.5 (45.4)	5>2, 3, 4>1
Percentage of psychosocial professionals with fixed–term contract	51.7 (43.0)	59.1 (42.3)	58.5 (40.5)	47.5 (42.8)	35.8 (43.8)	16.1 (32.4)	1, 2>3>4>5
Percentage of psychosocial professionals hired with SEP funds	22.3 (32.7)	18.6 (29.8)	25.7 (32.4)	28.5 (37.4)	9.9 (26.7)	0.0 (0.0)	2, 3>1>4>5
Percentage of psychosocial professionals hired with PIE funds	25.6 (35.1)	34.2 (39.1)	28.1 (34.4)	19.6 (31.3)	13.0 (29.0)	2.5 (15.3)	1>2>3>4>5

*One–way ANOVA tests were performed. The numbers in parentheses in column heads refer to the numbers used for illustrating significant differences in the post-hoc column*.

### Linear Multilevel Regressions and Quantile Regressions Analyses Predicting Math and Language 2017 SIMCE Test Scores

Table 4 shows the results of the multilevel estimation predicting math and language achievement score of eighth- and 10th-grade students in Model 1. With respect to the individual-level variables, findings showed that being a female student predicted lower scores in math in eighth (*b* = −6.71, *p* < 0.001) and 10th (*b* = −8.11, *p* < 0.001) grades and higher scores in language (*b* = 9.11 in eighth grade and *b* = 12.99 in 10th grade, *p* < 0.001). This is consistent with previous findings among Chilean students (Organisation for Economic Cooperation Development., 2012; Raczynski et al., 2013). Older students were associated with lower scores in both tests and grades (*b* = −13.48 to −8.64). On the other hand, a higher student SES predicted higher scores in math and language (*b* = 5.48 to 7.69). Ethnic origin only had a significant contribution to language scores in eighth grade (*b* = 0.93, *p* < 0.01) and to math scores in 10th grade (*b* = 0.93, *p* < 0.05). A higher student attendance rate (*b* = 0.18 to 1.07) and higher school motivation (*b* = 5.80 to 10.89) predicted higher scores in both language and math.

**Table 4:** Multilevel linear model predicting math and language score for 8th grade and 10th grade testing the contribution of the type of school according to hiring policy with individual and school–level predictors.

	**8th grade**		**10th grade**	
	**Math score**	**Language Score**	**Math score**	**Language score**
**Variables**	**b (SE)**	**b (SE)**	**b (SE)**	**b (SE)**
Individual level				
Female (Yes = 1)	−6.71^***^	9.95^***^	−8.11^***^	12.99^***^
	(0.21)	(0.24)	(0.30)	(0.28)
Age	−9.38^***^	−8.64^***^	−13.48^***^	−10.26^***^
	(0.17)	(0.19)	(0.24)	(0.23)
Socioeconomic status	7.42^***^	7.26^***^	7.69^***^	5.48^***^
	(0.17)	(0.19)	(0.23)	(0.21)
Indigenous ancestry	0.27	0.93^**^	0.93^*^	0.42
	(0.31)	(0.35)	(0.46)	(0.43)
Attendance	0.65^***^	0.18^***^	1.07^***^	0.40^***^
	(0.02)	(0.02)	(0.03)	(0.03)
School motivation	5.80^***^	6.89^***^	10.89^***^	8.31^***^
	(0.16)	(0.18)	(0.21)	(0.20)
School level				
Percentage of female students	0.15^***^	0.11^***^	0.19^***^	0.15^***^
	(0.02)	(0.02)	(0.03)	(0.02)
School SES (reference category: Low–SES school)				
Mid–low	7.23^***^	4.61^***^	14.59^***^	7.01^***^
	(0.79)	(0.72)	(1.65)	(1.22)
Mid	18.77^***^	12.52^***^	36.21^***^	16.95^***^
	(0.92)	(0.83)	(1.82)	(1.36)
Mid–high	32.17^***^	19.96^***^	52.98^***^	23.61^***^
	(1.14)	(1.03)	(2.13)	(1.59)
High	44.14^***^	23.80^***^	68.34^***^	31.94^***^
	(2.67)	(2.34)	(4.56)	(3.40)
Attendance (school average)	1.13^***^	1.30^***^	1.82^***^	1.54^***^
	(0.07)	(0.07)	(0.12)	(0.09)
School funding (reference category: public)				
Subsidized private	4.71^***^	0.05	1.67	−2.75^*^
	(0.70)	(0.62)	(1.51)	(1.11)
Private	17.61^***^	6.47^**^	4.97	−4.54
	(2.76)	(2.40)	(4.55)	(3.38)
Rural school (Yes = 1)	0.05	1.51^*^	−8.74^**^	−4.37^*^
	(0.84)	(0.76)	(2.71)	(2.05)
Type of school (reference category: No psychosocial professionals hired)				
Psychologists only	1.92^*^	−0.42	5.20^***^	1.48
	(0.76)	(0.68)	(1.35)	(1.00)
Social workers only	2.51	0.60	1.09	−1.13
	(2.11)	(1.86)	(3.37)	(2.48)
Psychosocial pairs	1.61	−1.93^*^	5.00^**^	0.52
	(0.85)	(0.75)	(1.61)	(1.19)
Constant	192.49^***^	202.76^***^	174.00^***^	211.47^***^
	(7.26)	(6.90)	(11.54)	(9.05)
Number of students	147,531	147,531	106,347	106,347
Number of schools	5,608	5,608	2,382	2,382
Log–likelihood	−749,854.2	−768,088.4	−558,834.0	−551,571.6

*Unstandardized coefficients reported. Standard errors in parentheses*.

* *p < 0.05*,

** *p < 0.01*,

*** *p < 0.001*.

When considering the school-level variables, the proportion of female students was related to higher scores in both math and language (*b* = 0.11 to 0.19). Higher school-level SES predicted better language and math achievement scores, with higher effects found in high-SES schools (*b* = 23.80 to 68.34). A higher average school attendance rate was associated with higher math and language scores (*b* = 1.13 to 1.82). With respect to school funding, there were only consistent contributions in the eighth-grade sample, wherein subsidized private schools (*b* = 4.71, *p* < 0.001) and private schools (*b* = 17.61, *p* < 0.001) had higher scores in the math SIMCE test; a similar effect was found for eighth-grade students from private schools (*b* = 6.47, *p* < 0.01) in language. Urbanicity had diverse effects on test scores, with a rural setting making a small but positive contribution to math scores in the 10th-grade sample (*b* = 1.51, *p* < 0.05) and a negative effect on language scores for eighth-grade (*b* = −8.74, *p* < 0.01) and 10th-grade (*b* = −4.37, *p* < 0.05) students.

With respect to the variables related to psychosocial professionals in Model 1, findings showed that schools that only hired psychologists predicted better math scores in eighth grade (*b* = 1.92, *p* < 0.05) and 10th grade (*b* = 5.20, *p* < 0.001) compared to schools without psychosocial professionals. Schools that only hired social workers did not have statistically significant contributions to test scores. Schools with psychosocial pairs did not show consistent effects, predicting lower language scores in eighth grade (*b* = −1.93, *p* < 0.05) and higher math scores in 10th grade (*b* = 5.00, *p* < 0.01).

Quantile regressions on the 25th, 50th, 75th, and 90th quantiles of performance were performed based on Model 1. Table 5 shows the results on math and language achievement of eighth grade students. In math, the presence of psychologist (*b* = 1.67, *p* < 0.01) and psychosocial pairs (*b* = 1.15, *p* < 0.05) predicted higher math scores for the 25th quantile group. On the contrary, the presence of social workers (*b* = −5.86, *p* < 0.05) and psychosocial pairs (*b* = −1.43, *p* < 0.001) predicted lower scores in the 90th quantile group. In language, the presence of psychologists predicted lower scores in all quantiles but the 90th (*b* = −1.07 to −1.47, *p* < 0.01). The presence of social workers predicted lower scores in the 50th (*b* = −2.80, *p* < 0.05) and 75th (*b* = −3.05, *p* < 0.05) quantiles. Finally, the presence of psychosocial pairs predicted lower scores in all quantiles (*b* = −1.30 to −3.21, *p* < 0.001 and *p* < 0.01).

**Table 5:** Quantile regression predicting math and language score for 8th grade testing the contribution of the type of school according to hiring policy with individual and school–level predictors.

	**Math Test**				**Language Test**			
**Variables**	**b (SE)**	**b (SE)**	**b (SE)**	**b (SE)**	**b (SE)**	**b (SE)**	**b (SE)**	**b (SE)**
Individual level									
Female (Yes = 1)	−6.67^***^	−7.27^***^	−7.39^***^	−7.25^***^	10.97^***^	10.02^***^	9.03^***^	7.87^***^
	(0.36)	(0.26)	(0.38)	(0.26)	(0.50)	(0.26)	(0.39)	(0.30)
Age	−10.19^***^	−10.54^***^	−10.56^***^	−10.35^***^	−9.15^***^	−9.50^***^	−9.87^***^	−9.08^***^
	(0.25)	(0.32)	(0.25)	(0.24)	(0.22)	(0.13)	(0.18)	(0.22)
Socioeconomic status	9.45^***^	10.02^***^	9.90^***^	9.55^***^	7.68^***^	9.07^***^	9.19^***^	8.58^***^
	(0.32)	(0.17)	(0.26)	(0.28)	(0.33)	(0.26)	(0.30)	(0.38)
Indigenous ancestry	−0.71	−0.35	−0.32	−0.37	1.85^***^	1.70^***^	1.03	0.69
	(0.50)	(0.25)	(0.50)	(0.26)	(0.43)	(0.46)	(0.71)	(0.86)
Attendance	0.47^***^	0.55^***^	0.71^***^	0.81^***^	0.17^***^	0.19^***^	0.14^**^	0.14^**^
	(0.03)	(0.03)	(0.03)	(0.04)	(0.03)	(0.03)	(0.04)	(0.05)
School motivation	5.08^***^	6.49^***^	7.62^***^	8,68^***^	7.87^***^	7.94^***^	7.65^***^	6.34^***^
	(0.33)	(0.17)	(0.29)	(0.28)	(0.21)	(0.23)	(0.26)	(0.18)
School level								
Percentage of female students	0.12^***^	0.12^***^	0.12^***^	0.09^***^	0.09^***^	0.11^***^	0.09^***^	0.07^***^
	(0.01)	(0.01)	(0.01)	(0.01)	(0.01)	(0.01)	(0.01)	(0.01)
School SES (reference category: Low–SES school)								
Mid–low	6.99^***^	7.62^***^	8.33^***^	9.02^***^	3.20^***^	5.01^***^	6.73^***^	7.79^***^
	(0.16)	(0.15)	(0.66)	(0.54)	(0.49)	(0.47)	(0.60)	(0.88)
Mid	18.21^***^	20.47^***^	21.20^***^	21.58^***^	12.04^***^	14.95^***^	17.15^***^	17.58^***^
	(0.38)	(0.44)	(1.01)	(0.91)	(0.48)	(0.65)	(0.78)	(0.71)
Mid–high	32.66^***^	33.62^***^	33.81^***^	34.18^***^	21.32^***^	24.12^***^	25.41^***^	24.42^***^
	(0.37)	(0.62)	(0.90)	(1.13)	(0.17)	(0.57)	(0.59)	(0.66)
High	44.99^***^	43.57^***^	41.02^***^	40.71^***^	25.03^***^	27.50^***^	25.33^***^	24.96^***^
	(1.05)	(0.83)	(1.22)	(1.13)	(1.84)	(2.28)	(1.67)	(1.33)
Attendance (school average)	1.42^***^	1.57^***^	1.65^***^	1.67^***^	1.62^***^	1.76^***^	1.64^***^	1.44^***^
	(0.06)	(0.09)	(0.05)	(0.02)	(0.07)	(0.08)	(0.03)	(0.04)
School funding (reference category: public)								
Subsidized private	5.11^***^	3.63^***^	2.70^***^	1.46^***^	−1.51^***^	−2.33	−3.50^***^	−3.44^***^
	(0.30)	(0.35)	(0.51)	(0.36)	(0.41)	(0.38)	(0.25)	(0.37)
Private	15.12^***^	13.59^***^	13.80^***^	8.90^***^	4.40^**^	2.34	1.55^***^	−0.69
	(0.80)	(1.16)	(0.54)	(2.25)	(1.67)	(2.13)	(1.91)	(0.69)
Rural school (Yes = 1)	−2.16^***^	−1.38^***^	−1.78^***^	−2.37^***^	0.26	−0.25	−0.80^*^	−1.93^**^
	(0.71)	(0.35)	(0.52)	(0.70)	(0.71)	(0.42)	(0.37)	(0.74)
Type of school (reference category: No psychosocial professionals hired)								
Psychologists only	1.67^**^	0.90	0.55	−0.58	−1.47^**^	−1.07^**^	−1.35^**^	−0.60
	(0.50)	(0.46)	(0.36)	(0.30)	(0.54)	(0.32)	(0.50)	(0.48)
Social workers only	1.09	−0.40	−2.93	−5.86^*^	−1.26	−2.80^*^	−3.05^*^	−0.73
	(0.74)	(0.84)	(1.13)	(2.24)	(1.68)	(1.29)	(1.24)	(1.54)
Psychosocial pairs	1.15^*^	0.58	0.48	−1.43^***^	−3.21^***^	−2.93^***^	−2.64^***^	−1.30^**^
	(0.56)	(0.41)	(0.27)	(0.41)	(0.40)	(0.42)	(0.56)	(0.45)
Constant	169.90^***^	182.09^***^	189.55^***^	205.09^***^	154.54^***^	174.02^***^	226.35^***^	261.68^***^
	(4.63)	(9.17)	(4.89)	(4.41)	(7.01)	(6.43)	(5.27)	(7.07)
Number of students	147,880	147,880	147,880	147,880	147,880	147,880	147,880	147,880
Pseudo R2	0.1434	0.1538	0.1553	0.1529	0.0792	0.0907	0.0891	0.0789

*Unstandardized coefficients reported. Robust standard errors in parentheses*.

* *p < 0.05*,

** *p < 0.01*,

*** *p < 0.001*.

Table 6 presents the results of the quantile regressions on math and language in tenth grade students Similar to eight graders, schools that only hired psychologists predicted higher scores in math tests in all quantiles but the 50th quantile (*b* = 1.12 to 1.85, *p* < 0.001 and *p* < 0.05), but did not had statistically significant effects on language scores. Schools with only social workers predicted lower scores in math (*b* = −3.14 to −4.19, *p* < 0.05) and language (*b* = −2.94 to −6.74, *p* < 0.001) in all quantiles but the 90th. Finally, schools that hired psychosocial pairs did not have statistically significant effects on math scores but predicted lower language scores on the 75th and 90th quantiles (*b* = −1.53 to −1.13, *p* < 0.05 and *p* < 0.001). Wald tests were performed after estimation to test whether the magnitude of the coefficients was different between quantiles, but the equality hypothesis was not rejected at the 5% level.

**Table 6:** Quantile regression predicting math and language score for 10th grade testing the contribution of the type of school according to hiring policy with individual and school–level predictors.

	**Math Test**				**Language Test**			
**Variables**	**b (SE)**	**b (SE)**	**b (SE)**	**b (SE)**	**b (SE)**	**b (SE)**	**b (SE)**	**b (SE)**
Individual level								
Female (Yes = 1)	−7.53^***^	−8.80^***^	−9.83^***^	−11.06^***^	16.82^***^	13.15^***^	8.95^***^	7.18^***^
	(0.41)	(0.47)	(0.49)	(0.47)	(0.46)	(0.15)	(0.29)	(0.42)
Age	−16.85^***^	−15.97^***^	−14.91^***^	−13.63^***^	−11.72^***^	−12.41^***^	−12.13^***^	−11.39^***^
	(0.44)	(0.24)	(0.14)	(0.28)	(0.36)	(0.29)	(0.33)	(0.41)
Socioeconomic status	10.81^***^	9.78^***^	8.73^***^	8.27^***^	5.79^***^	6.93^***^	7.16^***^	7.40^***^
	(0.29)	(0.28)	(0.15)	(0.37)	(0.26)	(0.32)	(0.32)	(0.06)
Indigenous ancestry	0.79	0.94	−0.05	−1.92^***^	1.15^*^	0.55	0.06	−0.45
	(0.52)	(0.63)	(0.84)	(0.48)	(0.58)	(0.37)	(0.45)	(1.04)
Attendance	0.95^***^	0.99^***^	1.07^***^	1.11^***^	0.35^***^	0.38^***^	0.36^***^	0.34^***^
	(0.03)	(0.03)	(0.02)	(0.05)	(0.03)	(0.02)	(0.02)	(0.02)
School motivation	11.83^***^	12.01^***^	12.39^***^	12.06^***^	10.23^***^	9.63^***^	9.11^***^	8.10^***^
	(0.27)	(0.20)	(0.16)	(0.27)	(0.38)	(0.28)	(0.30)	(0.33)
School level								
Percentage of female students	0.17^***^	0.12^***^	0.08^***^	0.06^***^	0.14^***^	0.10^***^	0.09^***^	0.06^***^
	(0.01)	(0.01)	(0.01)	(0.01)	(0.01)	(0.01)	(0.01)	(0.01)
School SES (reference category: Low–SES school)									
Mid–low	13.43^***^	17.43^***^	20.08^***^	21.22^***^	6.16^***^	8.83^***^	11.37^***^	12.90^***^
	(0.73)	(0.75)	(1.08)	(0.65)	(0.60)	(0.82)	(0.78)	(0.92)
Mid	37.49^***^	41.93^***^	40.14^***^	37.36^***^	17.86^***^	20.58^***^	23.59^***^	23.92^***^
	(1.18)	(0.55)	(0.92)	(1.09)	(0.84)	(0.84)	(0.95)	(0.95)
Mid–high	58.71^***^	57.21^***^	52.24^***^	47.45^***^	25.38^***^	27.20^***^	29.80^***^	29.17^***^
	(1.23)	(0.69)	(1.20)	(1.33)	(0.53)	(0.63)	(0.98)	(0.97)
High	70.42^***^	66.70^***^	59.34^***^	56.57^***^	27.64^***^	32.96^***^	32.63^***^	34.06^***^
	(1.41)	(1.34)	(2.41)	(4.26)	(1.00)	(1.44)	(1.16)	(3.18)
Attendance (school average)	2.25^***^	2.31^***^	2.30^***^	2.28^***^	1.92^***^	1.94^***^	1.80^***^	1.64^***^
	(0.07)	(0.05)	(0.02)	(0.06)	(0.03)	(0.03)	(0.03)	(0.08)
School funding (reference category: public)									
Subsidized private	−1.23	−3.84^***^	−5.48	−6.06^***^	−4.92^***^	−6.63^***^	−8.26^***^	−9.41^***^
	(0.77)	(0.60)	(0.37)	(0.67)	(0.52)	(0.35)	(0.41)	(0.45)
Private	4.35^*^	−0.00	−0.55	−2.81	−1.54	−5.71^***^	−4.37^***^	−9.15^**^
	(2.04)	(1.97)	(1.60)	(3.64)	(1.06)	(0.37)	(0.27)	(3.14)
Rural school (Yes = 1)	−8.02^***^	−9.45^***^	−8.53^***^	−10.10^***^	−2.98^**^	−2.20	−2.04^*^	−2.42
	(1.97)	(1.42)	(1.25)	(2.13)	(1.02)	(1.71)	(1.00)	(2.26)
Type of school (reference category: No psychosocial professionals hired)								
Psychologists only	1.30^*^	1.13	1.12^***^	1.85^***^	0.28	0.19	−0.53	−0.36
	(0.66)	(0.65)	(0.29)	(0.31)	(0.56)	(0.56)	(0.36)	(0.74)
Social workers only	−4.19^*^	−4.00^*^	−3.14^*^	−1.17	−6.74^***^	−5.41^***^	−2.94^***^	−2.38
	(1.81)	(1.56)	(1.54)	(1.90)	(0.50)	(0.77)	(0.66)	(1.24)
Psychosocial pairs	2.13	1.30	0.59	0.74	−1.44	−0.98	−1.13^*^	−1.53^***^
	(1.44)	(0.79)	(0.57)	(0.96)	(0.91)	(1.02)	(0.44)	(0.35)
Constant	173.69^***^	189.84^***^	204.43^***^	213.61	175.51^***^	215.97^***^	258.47^***^	293.12^***^
	(13.03)	(5.78)	(3.74)	(5.78)	(10.81)	(5.82)	(7.42)	(10.56)
Number of students	106,347	106,347	106,347	106,347	106,347	106,347	106,347	106,347
Pseudo R2	0.2076	0.2208	0.1991	0.1771	0.1197	0.1246	0.1213	0.1087

*Unstandardized coefficients reported. Robust standard errors in parentheses*.

* *p < 0.05*,

** *p < 0.01*,

*** *p < 0.001*.

The results of the estimation of Model 2 are reported in Table 7. In Model 2, the variable related to the type of school according to its hiring policy was replaced by variables reporting the number of psychologists and social workers hired by a school. To provide a better understanding of the relationship of these variables with school SES, we introduced a different index that reflects the proportion of vulnerable students and the moderation effect with the number of psychosocial professionals hired. Findings showed that the proportion of vulnerable students predicted lower scores in math and language (*b* = −0.25 to −1.39) and number of psychologists hired in the schools had a positive effect in the scores of math tests in eighth grade (*b* = 1.84, *p* < 0.001) and 10th grade (*b* = 1.55, *p* < 0.001), with no significant effects on language scores. The interaction effects showed that the positive direct effect found for the number of psychologists hired was moderated by the proportion of vulnerable students for the eighth-grade sample (*b* = −0.08 to −0.05), reducing their effectiveness in schools with more vulnerable students. In contrast, the number of social workers was related to negative effects on language and math scores (*b* = −2.64 to −8.06). However, the moderation effect between the number of social workers and the proportion of vulnerable students predicted a positive effect in all tests but language for the 10th-grade sample (*b* = 0.07 to 0.36), meaning that a higher number of social workers had a positive effect on school achievement in schools with a greater proportion of low-SES students.

**Table 7:** Multilevel linear model predicting math and language score for 8th grade and 10th grade testing the contribution of the number of psychosocial professionals hired with individual and school–level predictors.

	**8th grade**		**10th grade**	
	**Math score**	**Language Score**	**Math score**	**Language score**
**Variables**	**b (SE)**	**b (SE)**	**b (SE)**	**b (SE)**
Individual level				
Female (Yes = 1)	−6.68^***^	9.99^***^	−8.09^***^	13.01^***^
	(0.21)	(0.24)	(0.30)	(0.28)
Age	−9.44^***^	−8.71^***^	−13.51^***^	−10.30^***^
	(0.17)	(0.19)	(0.24)	(0.23)
Socioeconomic status	7.90^***^	7.82^***^	8.06^***^	5.81^***^
	(0.17)	(0.19)	(0.23)	(0.21)
Indigenous ancestry	0.33	0.89^*^	0.96^*^	0.40
	(0.31)	(0.35)	(0.46)	(0.43)
Attendance	0.64^***^	0.17^***^	1.07^***^	0.39^***^
	(0.02)	(0.02)	(0.03)	(0.03)
School motivation	5.76^***^	6.83^***^	10.87^***^	8.29^***^
	(0.16)	(0.18)	(0.21)	(0.20)
School level				
Percentage of female students	0.19^***^	0.13^***^	0.20^***^	0.16^***^
	(0.02)	(0.02)	(0.04)	(0.03)
Attendance (school average)	1.40^***^	1.46^***^	2.53^***^	1.90^***^
	(0.08)	(0.07)	(0.13)	(0.09)
School funding (reference category: public)				
Subsidized private	8.19^***^	2.57^***^	1.51	−2.82^*^
	(0.70)	(0.61)	(1.65)	(1.17)
Private	39.74^***^	17.19^***^	30.09^***^	8.02^***^
	(1.51)	(1.31)	(2.57)	(1.84)
Rural school (Yes = 1)	0.89	1.13	−6.63^*^	−4.47^*^
	(0.91)	(0.82)	(2.94)	(2.14)
Number of psychologists hired	1.84^***^	0.22	1.55^**^	0.20
	(0.33)	(0.28)	(0.56)	(0.39)
Number of social workers hired	−4.76^***^	−2.64^***^	−8.06^***^	−3.06^***^
	(0.64)	(0.55)	(1.26)	(0.89)
Percentage of vulnerable students	−0.48^***^	−0.25^***^	−1.39^***^	−0.52^***^
	(0.03)	(0.03)	(0.10)	(0.07)
Moderation effects				
Number of psychologists x % Vulnerable students	−0.08^***^	−0.05^*^	−0.05	−0.02
	(0.02)	(0.02)	(0.06)	(0.04)
Number of social workers x % Vulnerable students	0.20^***^	0.07^**^	0.36^***^	0.05
	(0.03)	(0.03)	(0.08)	(0.06)
Constant	186.48^***^	200.33^***^	150.78^***^	197.94^***^
	(7.47)	(7.02)	(12.12)	(9.20)
Number of students	147,531	147,531	106,347	106,347
Number of schools	5,608	5,608	2,382	2,382
Log–likelihoodd	−751,860.8	−770,046.3	−559,016.2	−551,652

*Unstandardized coefficients reported. Standard errors in parentheses*.

* *p < 0.05*,

** *p < 0.01*,

*** *p < 0.001*.

Additional estimations that took the form of robustness checks were conducted using different measures of the presence of psychosocial professionals in schools described as Models 3 and 4 (see Supplementary Tables 1, 2). Results of these models predicted that the type of contract of professionals hired did not had significant effects on achievement, and that a higher proportion of psychosocial professionals hired with SEP funds was associated with better math scores among eighth-grade students; and a higher proportion of psychosocial professionals hired with PIE funds was associated with lower scores in math and language.

### Logistic Multilevel Models Predicting Short- and Long-Term School Dropout

Table 8 presents the logistic multilevel model predicting students' dropout for the sample of eighth-grade students. Columns A and B of each model show the effects of independent variables on short- and long-term dropout, respectively. The predictors in the estimations of the probability of dropping out were the same used in the estimation of math and language test scores. The odds ratios (ORs) were calculated by exponentiating the coefficients obtained from the estimations. ORs compare the relative odds of occurrence of dropout given the exposure to a predictor. If an OR is > 1, the exposure to a predictor is associated with higher odds of dropping out; if it is lower than 1, it is associated with lower odds.

**Table 8:** Multilevel logistic model predicting the probability of students' short and long–term dropout for 8th grade with individual and school–level predictors.

	**Model 1 (A)**	**Model 1 (B)**	**Model 2 (A)**	**Model 2 (B)**	**Model 3 (A)**	**Model 3 (B)**	**Model 4 (A)**	**Model 4 (B)**
**Variables**	**OR (SE)**	**OR (SE)**	**OR (SE)**	**OR (SE)**	**OR (SE)**	**OR (SE)**	**OR (SE)**	**OR (SE)**
Individual level								
Female (Yes = 1)	0.97	0.97	0.98	0.97	0.98	0.97	0.98	0.96
	(0.05)	(0.06)	(0.05)	(0.06)	(0.06)	(0.06)	(0.06)	(0.06)
Age	3.97^***^	4.21^***^	3.97^***^	4.21^***^	3.98^***^	4.19^***^	4.01^***^	4.21^***^
	(0.10)	(0.11)	(0.10)	(0.11)	(0.11)	(0.12)	(0.11)	(0.12)
Socioeconomic status	1.12	1.13	1.12	1.13	1.13	1.15	1.13	1.15
	(0.11)	(0.11)	(0.11)	(0.11)	(0.11)	(0.11)	(0.11)	(0.11)
Indigenous ancestry	0.77^**^	0.78^**^	0.77^**^	0.78^**^	0.79^**^	0.77^**^	0.79^**^	0.77^**^
	(0.06)	(0.06)	(0.06)	(0.06)	(0.07)	(0.07)	(0.07)	(0.07)
Attendance	0.95^***^	0.95^***^	0.95^***^	0.95^***^	0.94^***^	0.95^***^	0.95^***^	0.95^***^
	(0.00)	(0.00)	(0.00)	(0.00)	(0.00)	(0.00)	(0.00)	(0.00)
School motivation	1.13	1.18^***^	1.13	1.18^***^	1.14	1.18^***^	1.13	1.18^***^
	(0.10)	(0.05)	(0.10)	(0.05)	(0.10)	(0.05)	(0.10)	(0.05)
School level								
Percentage of female students	0.99^**^	0.99^*^	0.99^**^	0.99^*^	0.99^*^	1.00^*^	0.99^*^	0.99^*^
	(0.00)	(0.00)	(0.00)	(0.00)	(0.00)	(0.00)	(0.00)	(0.00)
School SES (reference category: Low–SES school)								
Mid–low	0.90	0.91	0.91	0.92	0.93	0.93	0.93	0.94
	(0.09)	(0.09)	(0.09)	(0.09)	(0.09)	(0.09)	(0.09)	(0.10)
Mid	0.87	0.90	0.88	0.92	0.98	1.02	0.98	1.02
	(0.10)	(0.10)	(0.10)	(0.10)	(0.11)	(0.12)	(0.11)	(0.12)
Mid–high	0.61^**^	0.55^***^	0.63^**^	0.56^***^	0.67^*^	0.61^**^	0.65^*^	0.60^**^
	(0.09)	(0.09)	(0.09)	(0.09)	(0.11)	(0.11)	(0.11)	(0.11)
High	0.48	0.31^*^	0.49	0.32^*^	0.36	0.17^*^	0.22	0.00
	(0.20)	(0.15)	(0.20)	(0.15)	(0.22)	(0.13)	(0.17)	(0.00)
Attendance (school average)	0.94^***^	0.94^***^	0.94^***^	0.94^***^	0.94^***^	0.94^***^	0.95^***^	0.94^***^
	(0.01)	(0.01)	(0.01)	(0.01)	(0.01)	(0.01)	(0.01)	(0.01)
School funding (reference category: public)								
Subsidized private	0.70^***^	0.69^***^	0.70^***^	0.70^***^	0.68^***^	0.66^***^	0.67^***^	0.65^***^
	(0.06)	(0.06)	(0.06)	(0.06)	(0.05)	(0.05)	(0.05)	(0.05)
Private	0.99	1.32	1.05	1.42	1.52	2.53		
	(0.40)	(0.61)	(0.42)	(0.65)	(0.93)	(1.94)		
Rural school (Yes = 1)	0.85	0.85	0.83	0.83	0.84	0.86	0.83	0.85
	(0.10)	(0.10)	(0.10)	(0.10)	(0.10)	(0.11)	(0.10)	(0.11)
Type of school (reference category: No psychosocial professionals hired)								
Psychologists only	0.76^**^	0.76^**^						
	(0.07)	(0.08)						
Social workers only	0.49^*^	0.50^*^						
	(0.14)	(0.15)						
Psychosocial pairs	0.66^***^	0.66^***^						
	(0.07)	(0.07)						
Number of psychologists hired			0.94^*^	0.94^*^				
			(0.03)	(0.03)				
Number of social workers hired			0.88^*^	0.89^*^				
			(0.04)	(0.05)				
Percentage of psychosocial professionals with indefinite contract					1.00	1.00		
					(0.00)	(0.00)		
Percentage of psychosocial professionals with fixed–term contract					1.00	1.00		
					(0.00)	(0.00)		
Percentage of psychosocial professionals hired with SEP funds							1.00	1.00
							(0.00)	(0.00)
Percentage of psychosocial professionals hired with PIE funds							1.00	1.00
							(0.00)	(0.00)
Constant	0.00^***^	0.00^***^	0.00^***^	0.00^***^	0.00^***^	0.00^***^	0.00^***^	0.00^***^
	(0.00)	(0.00)	(0.00)	(0.00)	(0.00)	(0.00)	(0.00)	(0.00)
Number of students	147,531	147,531	147,531	147,531	123,574	123,574	118,239	118,239
Number of schools	5,608	5,608	5,608	5,608	4,536	4,536	4,410	4,410
Log–likeli~d	−7,810.49	−7010.19	−7811.10	−7011.40	−6569.71	−5935.74	−6360.62	−5774.97

*Standard errors in parentheses. Columns A used the short–term dropout as dependent variable. Columns B used the long–term dropout as dependent variable*.

* p < 0.05;

** p < 0.01;

*** *p < 0.001*.

Regarding individual-level variables, students' gender and SES were not statistically significant in this sample. Being an older student was associated with higher odds of dropping out (*OR* = 3.97 to 4.21, *p* < 0.001). Students with an Indigenous background had OR below 1 in all models, predicting that these students were less likely to drop out (*OR* = 0.77 to 0.79, *p* < 0.001). A higher attendance predicted lower odds of dropout in the short and long term (*OR* = 0.94 to 0.95, *p* < 0.001). School motivation did not show a statistically significant effect in any model using the short-term dropout variable, but it had a counterintuitive effect in all models of long-term dropout, wherein higher motivation predicted higher odds of dropping out (*OR* = 1.18, *p* < 0.001).

Regarding school-level variables, the proportion of female students in schools predicted a lower likelihood of students dropping out. Higher attendance predicted lower odds of dropout in the short and long term (*OR* = 0.98 to 0.99, *p* < 0.001). School SES was linked with statistically significant differences between medium-high SES schools and low-SES schools, with the former predicting lower odds of dropout (*OR* = 0.64 to 0.69, *p* < 0.001). Rural schools did not make a significant contribution in any estimated models. With respect to type of school, private subsidized schools predicted a lower probability of having students that dropped out compared to public schools (*OR* = 3.98 to 4.21, *p* < 0.001).

Considering the relevant study variables at the school level, schools with only psychologists reduced the odds of short-term (*OR* = 0.76, *p* < 0.05) and long-term (*OR* = 0.76, *p* < 0.01) dropout compared to schools with no psychosocial professionals hired. Schools with only social workers also predicted lower odds of short-term (*OR* = 0.49, *p* < 0.05) and long-term (*OR* = 0.50, *p* < 0.05) dropout, and schools with psychosocial pairs hired predicted a lower likelihood of students dropping out in the same year (*OR* = 0.66, *p* < 0.001) and in the following 2 years (*OR* = 0.66, *p* < 0.001). A higher number of psychologists hired reduced the odds of dropping out (short term: *OR* = 0.94, *p* < 0.05; long term: *OR* = 0.94, *p* < 0.05). Similar results were found for the number of social workers hired, wherein a higher number reduced the probability of students dropping out in the short term (*OR* = 0.88, *p* < 0.010) and long term (*OR* = 0.89, *p* < 0.01). The contributions of the proportion of professionals hired based on the type of contract and funds used were not statistically significant in the sample of eighth-grade students.

Table 9 reports the results of the logistic multilevel estimations for the sample of 10th-grade students. Regarding individual-level variables, being a female student predicted lower odds of dropout in all models (*OR* = 0.65 to 0.68, *p* < 0.001). Age had similar effects in the eighth-grade sample, wherein older students were associated with a higher probability of dropping out of school in the short and long term (*OR* = 4.42 to 4.65, *p* < 0.001). Attendance predicted a lower likelihood of dropping out in the short and long term (*OR* = 0.91 to 0.92, *p* < 0.001). In contrast to findings for eighth-grade students, school motivation in this sample was statistically significant in all models, wherein higher motivation was related with a lower likelihood of dropout (*OR* = 0.70 to 0.72, *p* < 0.001). Indigenous background and individual SES were not statistically significant for this sample.

**Table 9:** Multilevel logistic model predicting the probability of students' short and long–term dropout for 10th grade with individual and school–level predictors.

	**Model 1 (A)**	**Model 1 (B)**	**Model 2 (A)**	**Model 2 (B)**	**Model 3 (A)**	**Model 3 (B)**	**Model 4 (A)**	**Model 4 (B)**
**Variables**	**OR (SE)**	**OR (SE)**	**OR (SE)**	**OR (SE)**	**OR (SE)**	**OR (SE)**	**OR (SE)**	**OR (SE)**
Individual level								
Female (Yes = 1)	0.68^***^	0.66^***^	0.68^***^	0.66^***^	0.67^***^	0.65^***^	0.66^***^	0.65^***^
	(0.03)	(0.03)	(0.03)	(0.03)	(0.03)	(0.03)	(0.03)	(0.03)
Age	4.51^***^	4.65^***^	4.51^***^	4.65^***^	4.42^***^	4.58^***^	4.44^***^	4.59^***^
	(0.11)	(0.12)	(0.11)	(0.12)	(0.12)	(0.13)	(0.12)	(0.13)
Socioeconomic status	1.01	1.01	1.01	1.01	1.01	1.01	1.01	1.02
	(0.03)	(0.03)	(0.03)	(0.03)	(0.04)	(0.04)	(0.04)	(0.04)
Indigenous ancestry	0.92	0.92	0.92	0.92	0.91	0.91	0.91	0.91
	(0.06)	(0.06)	(0.06)	(0.06)	(0.07)	(0.07)	(0.07)	(0.07)
Attendance	0.91^***^	0.92^***^	0.91^***^	0.92^***^	0.92^***^	0.92^***^	0.92^***^	0.92^***^
	(0.00)	(0.00)	(0.00)	(0.00)	(0.00)	(0.00)	(0.00)	(0.00)
School motivation	0.72^***^	0.72^***^	0.72^***^	0.72^***^	0.71^***^	0.70^***^	0.70^***^	0.70^***^
	(0.02)	(0.02)	(0.02)	(0.02)	(0.03)	(0.03)	(0.03)	(0.03)
School level								
Percentage of female students	1.00	1.00	1.00	1.00	1.00	1.00	1.00	1.00
	(0.00)	(0.00)	(0.00)	(0.00)	(0.00)	(0.00)	(0.00)	(0.00)
School SES (reference category: Low–SES school)								
Mid–low	1.06	1.08	1.07	1.08	1.07	1.08	1.07	1.07
	(0.09)	(0.09)	(0.09)	(0.09)	(0.09)	(0.09)	(0.09)	(0.09)
Mid	0.83	0.83	0.84	0.83	0.82	0.81	0.82	0.81
	(0.08)	(0.08)	(0.08)	(0.08)	(0.09)	(0.09)	(0.09)	(0.09)
Mid–high	0.50^***^	0.48^***^	0.49^***^	0.48^***^	0.47^***^	0.44^***^	0.48^***^	0.45^***^
	(0.07)	(0.06)	(0.06)	(0.06)	(0.07)	(0.07)	(0.07)	(0.07)
High	1.04	0.84	1.02	0.82	0.87	0.80	0.85	0.79
	(0.32)	(0.28)	(0.32)	(0.27)	(0.34)	(0.32)	(0.34)	(0.33)
Attendance (school average)	0.99	0.99	0.99	0.99	0.99	0.99	0.99	0.99
	(0.01)	(0.01)	(0.01)	(0.01)	(0.01)	(0.01)	(0.01)	(0.01)
School funding (reference category: public)								
Subsidized private	1.10	1.08	1.10	1.09	1.13	1.12	1.14	1.13
	(0.08)	(0.08)	(0.08)	(0.08)	(0.09)	(0.09)	(0.09)	(0.09)
Private	0.38^**^	0.39^**^	0.40^**^	0.41^*^	0.43^*^	0.38^*^		
	(0.12)	(0.14)	(0.13)	(0.14)	(0.18)	(0.17)		
Rural school (Yes = 1)	0.93	0.93	0.93	0.92	0.95	0.96	0.97	0.98
	(0.16)	(0.16)	(0.16)	(0.16)	(0.17)	(0.17)	(0.17)	(0.17)
Type of school (reference category: No psychosocial professionals hired)								
Psychologists only	0.85^*^	0.84^*^						
	(0.07)	(0.07)						
Social workers only	0.92	0.90						
	(0.16)	(0.16)						
Psychosocial pairs	0.82^*^	0.79^*^						
	(0.07)	(0.07)						
Number of psychologists hired			0.95^*^	0.95^*^				
			(0.02)	(0.02)				
Number of social workers hired			0.97	0.97				
			(0.04)	(0.05)				
Percentage of psychosocial professionals with indefinite contract					1.00	1.00		
					(0.00)	(0.00)		
Percentage of psychosocial professionals with fixed–term contract					1.00	1.00		
					(0.00)	(0.00)		
Percentage of psychosocial professionals hired with SEP funds							1.00	1.00
							(0.00)	(0.00)
Percentage of psychosocial professionals hired with PIE funds							1.00	1.00
							(0.00)	(0.00)
Constant	0.00^***^	0.00^***^	0.00^***^	0.00^***^	0.00^***^	0.00^***^	0.00^***^	0.00^***^
	(0.00)	(0.00)	(0.00)	(0.00)	(0.00)	(0.00)	(0.00)	(0.00)
Number of students	106,347	106,347	106,347	106,347	85,636	85,636	80,773	80,773
Number of schools	2,382	2,382	2,382	2,382	1,824	1,824	1,702	1,702
Log–likelihood	−9,312.88	−9,039.37	−9,312.46	−9,039.57	−7,624.23	−7,391.42	−7,426.67	−7,230.20

*Standard errors in parentheses. Columns A used the short-term dropout as dependent variable. Columns B used the long-term dropout as dependent variable*.

p < 0.05;

** p < 0.01;

*** *p < 0.001*.

At the school level, the percentage of female students in school was not statistically significant in any model. Medium-high SES schools showed lower odds of dropout in all models (*OR* = 0.49 to 0.55, *p* < 0.001), compared to low-SES schools. Private schools predicted a lower likelihood of dropout compared to public schools in all models with this variable (*OR* = 0.38 to 0.43, *p* < 0.05 and *p* < 0.01).

Schools with only psychologists had lower odds of dropout in the short term (*OR* = 0.85, *p* < 0.05) and long term (*OR* = 0.84, *p* < 0.05). Schools with psychosocial pairs also had a lower likelihood of students dropping out in the short term (*OR* = 0.84, *p* < 0.050) and long term (*OR* = 0.81, *p* < 0.05) compared to schools with no psychosocial professionals hired. The number of psychologists hired predicted a lower probability of short-term (*OR* = 0.95, *p* < 0.05) and long-term (*OR* = 0.95, *p* < 0.05) dropout. The effects related to the number of social workers in the eighth-grade sample were not replicated in the sample of 10th-grade students. Similarly, the proportion of professionals hired with different contracts and funds did not have statistically significant effects on dropout.

## Discussion

Academic achievement and school dropout are considered indicators of school achievement and school failure, respectively. Both have been pinpointed as relevant indicators of educational quality worldwide (Organisation for Economic Cooperation Development., 2019). However, attaining the goal of quality education for all students (Ainscow, 2019) in an inclusive educational context has not been an easy task, and most countries are struggling with unequal distribution of gains and failures among students due to socioeconomic differences and related factors. In particular, school failure is overrepresented among poorer students and students with learning and behavioral difficulties at school. It has become a political and social problem, with well-known negative consequences for individuals and society such as the achievement gap and the school-to-prison pipeline (Ruiz et al., 2018; Granvik et al., 2020).

In this context, the presence of non-teaching professionals in the school may provide important support for students, especially for those who—due to factors related to the school and outside of the school—find it difficult to engage in the learning process. However, there is scarce scientific knowledge accumulated concerning the effect of school counselors, psychologists, social workers, and other professionals on issues such as retention and academic performance (López et al., 2017; Kuperminc et al., 2019; Arslan and Coşkun, 2020). Within this context, this study aimed to analyze the effects of school psychologist and social workers, when working together as “psychosocial pairs” or not, on relevant indicators of school achievement and failure.

Several conclusions can be drawn from the findings of this national study. First, schools seem to have a differentiated scheme for incorporating psychologists or social workers, based on certain characteristics of their students. Schools with a higher proportion of students from low- and mid-low SES tend to hire more social workers, and schools with a higher proportion of students from mid-low and mid-SES tend to hire more psychologists. Although high-SES schools tend to hire only psychologists who also have more indefinite contracts, low-SES schools tend to hire more psychosocial pairs with SEP and PIE funds, which are state funds for low-SES students and students with disabilities, respectively. These findings suggest that not only does the nature of the interventions that school psychologists implement differ disciplinarily, but also that these professionals are targeted by contract to different school populations. These findings require further exploration. On the other hand, these results can be linked to the intervention models attributed to each professional type. According to previous studies in Chile, social workers are associated with a network-and-benefits management model, in charge of providing support at the health and welfare level (Concha, 2012; Cádiz and Manriquez., 2015). School psychologists are associated with individual treatment of problems at an academic, emotional, and social level (Erausquin and Bur, 2013; López and Carrasco, 2018; Cárcamo-Vásquez et al., 2020). In this way, a higher proportion of social workers in more vulnerable schools may be due to the need for timely access to benefits and support for families that allow students to stay in school.

Secondly, findings regarding the contribution of school psychologists and social workers on students' performance in math and language tests are inconclusive. On the one hand, this study showed a positive association between the presence and number of psychologists and math achievement. In eighth grade, school psychologists (and psychosocial pairs) have a significant effect on math gains, particularly for lower-performing student. In tenth grade, the positive associations between psychologists and math scores are significant in all but one quantile. These results could be explained by the content of the interventions developed by school psychologists, which are generally associated with the development of social-emotional skills such as problem solving, emotional regulation, and social skills (Cárcamo-Vásquez et al., 2020). These interventions are developed as tier 2 or tier 3 interventions, that is, in groups of students or individually. Several studies have shown positive association between the development of social-emotional skills and mathematical achievement (Matthews et al., 2009; Prafitriyani et al., 2019; Slot et al., 2020). For example, Masitoh and Fitriyani (2018) have shown that problem-solving ability has positive effects on the perception of self-efficacy in mathematics. In this aspect, the abilities to solve problems of daily life and their reinforcement through school psychologists could have a direct incidence in a better predisposition to learn this subject. On the other hand, a positive relationship has been observed between the perception of self-efficacy and achievement in mathematics (Bandura, 1982; Poynton and Lapan, 2017; Rahmi et al., 2017). Similarly, Matthews et al. (2009) found a positive relationship between self-regulation and math achievement, visualizing that support in this area can also contribute in a substantive way. These results are encouraging regarding the impact that these interventions could have at level 1 and 2. However, future research needs to continue to build on these findings. However, there was also evidence, at least in eighth grade, of a negative association between the presence of psychologists and achievement in language. In this grade level, the presence of psychologists was associated with lower scores for all students except for very high achieving students. This negative association between the presence of psychologists and language achievement was not found in tenth grade. These findings also require further research.

On the other hand, findings regarding the associations between social workers and school achievement are complex in a different way, given the characteristics of the schools in which most social workers work. As noted, schools with low- and mid-low SES tend to hire more social workers, suggesting that schools require and ask them to work with students from lower-SES backgrounds. In some manner, this is a tiered support system in which the tier is not based on promotion or prevention strategies, but on students' SES and associated difficulties that, based on deficit theories, are assumed to require individual attention. These individual interventions are usually welfare-based strategies such as home visits (López and Carrasco, 2018). Findings from this study show that although the number of social workers in the total sample was related to negative effects on language and math scores, this was true only for schools with only social workers (which as we have shown is more frequent in lower-SES schools) and only for higher-achieving students in eighth grade, but for all but higher-achieving students in tenth grade. The associations were also moderated by school SES, wherein having more social workers positively affected school achievement in schools with a greater proportion of low-SES students. This moderation effect is highly important to consider. The negative effects on school achievement in the total sample might be due to the overrepresentation of social workers in low-SES schools. But even so, more social workers in low-SES schools might allow these social workers to not only work with more students but perhaps use more promotion and prevention (tier 1 and tier 2) strategies, which could explain why their greater presence positively affected student achievement in low-SES schools. However, it does not explain why the presence and number of social workers negatively affects higher-achieving student's performance. It might be due to an indirect effect on lower academic expectations on behalf of classroom teachers as an effect of a more network and welfare-based than academically-oriented school climate and support systems; however, this hypothesis requires further exploration.

Third, findings suggest that supporting students academically is naturally dependent on the type of work that psychosocial professionals are hired to do and with which students they are asked to work. In Chilean schools, these professionals are mainly hired through SEP or PIE funds. Whereas, the former provides more voucher support per student to schools that cater to lower-SES students identified as “socioeconomically vulnerable,” the latter offers more voucher funding per student officially diagnosed with a disability. Although both allow hiring of psychologists and social workers from the school, SEP's policy does not delimit an exclusive work with students belonging to SEP. On the contrary, PIE policy requires schools to attend to the needs of students with special educational needs previously diagnosed with a specific permanent or transitory disability. In practice, this means that the professionals financed by SEP have more freedom to diagnose, design and implement interventions than the professionals financed by PIE, who are required to develop a specialized diagnosis and develop a more individualized line of intervention. The fact that a higher proportion of psychosocial professionals hired with PIE funds are associated with lower math and language scores may therefore be due to the fact that these professionals are required to provide support exclusively to students with disabilities. In this regard, we suggest that futures studies analyze the impact of PIE-funded psychologists and social workers on students with and without diagnosed disabilities.

Fourth, findings regarding dropout as an indicator of school failure are promising. Schools that hired only psychologists, only social workers, or both (psychosocial pairs) had a reduced probability of students dropping out in the short and long term compared to schools that had no psychosocial professionals hired. In Chile, eighth grade is the last grade of primary school, and higher rates of school dropout occur between eighth and ninth grades, especially in schools that only provide primary schooling and oblige students to change schools. Similarly, hiring more psychologists and social workers was associated with a reduced likelihood of short- and long-term dropout for eighth-grade students. These are highly important findings that provide scientific evidence supporting public policies aimed at incorporating psychosocial professionals in the regular school system. However, these findings should be taken cautiously, considering evidence that these professionals are mainly performing tier 3 individual interventions with specific students and not implementing multi-tiered whole-school support systems (López et al., 2020).

Given the fact that in developing countries such as Chile, school psychologists and social workers are not required to hold graduate degrees to work in schools, not even professional diplomas related to school interventions, our findings suggest that with proper training on whole-school approaches and evidence-based intervention strategies to adequately meet the demands of the current inclusive educational context, these professionals could provide even more significant, relevant, and culturally sensitive supports for all students, with positive outcomes for students and school systems. Likewise, the lack of a clear intervention model and the emphasis on the development of individual intervention plans which are mandated by policies such as the PIE (Decree No. 170, 2010; Ministerio de Educación, 2016) and offered as suggestions by the SEP guidelines (Ministerio de Educación, 2017), makes it difficult to develop interventions that have an impact on the entire school community. In this aspect, we infer that the development of interventions at a level 1 and 2 can substantially contribute to the improvement of indicators such as performance and school dropout. During the COVID-19 pandemic, a promising venue has been the incorporation of a multi-tiered approach proposed by education researchers in april 2020 (Claro and Mizala, 2020) and later incorporated in the Chilean Ministry of Education's back to school guidelines (Ministerio de Educación, 2020b).

On the other hand, it is necessary to continue investigating the specific mechanisms through which school psychologists and social workers help to reduce dropout rates. Promotion and intervention strategies that support social and emotional learning, foster positive interpersonal relationships, develop a school mental health perspective, and improve school climate have been shown to increase students' sense of belonging to the school and academic and social success. These experiences have been shown to increase students' school commitment and attendance, factors that are critical to decreasing dropout rates (Hoagwood et al., 2007; Pate et al., 2016; Mason and Dye, 2017; Tello and Lonn, 2017; Filippello et al., 2019; Gubbels et al., 2019).

Overall, findings from this study support policies that increase funding for school psychologists and social workers, because their incorporation partly explains better school achievement and less school failure when controlling for individual and school characteristics. However, they highlight the need to further explore the mechanisms through which academic achievement and failure are developed with the support of psychologists and social workers in schools. These professionals, despite focusing most of their actions at level 3, have positive effects on students. This emphasizes the need to reformulate these actions from a whole-school perspective, opening the possibility of developing socioemotional skills in students via the curriculum and with greater participation of classroom teachers in tier 1 interventions (Hoagwood et al., 2007). As Mulhern (2020) and O'Connor (2018) pointed out, adults in the school are indispensable for the construction of a school climate that promotes a better school experience and achievement. However, they require time and space to develop these actions in schools.

In terms of policy and intervention, and to advance at a large scale a more evidence-based, tiered, whole-school approach, we suggest two strategies. First, the design and dissemination of training modules and comprehensive guidelines, particularly in the context of certified diplomas and master's-level training, could gradually be considered requisites for formal professional work in schools by non-teaching professional staff. Second, national or state-level policies should gradually require formal certification of training in school intervention approaches and the improvement of national and state-level datasets—in the case of Chile, the National Record of Educational Assistants—to allow registration of the multi-tiered type of interventions that school psychologists and social workers should be design and implement. This would improve opportunities for large-scale monitoring, follow up, and impact evaluations.

In this regard, a limitation of this study was the lack of a dataset from which to draw inferences regarding the tier or level of interventions developed by these professionals. Therefore, further studies should explore the effects of psychosocial professionals on student outcomes, considering the type of interventions deployed, and the proportion of professionals per school (O'Connor, 2018). Another limitation of the study was its cross-sectional design for the analyses on school achievement, which does not allow identifying causal relations between the study variables. A possible venue for future research is, therefore, to estimate such effects using longitudinal data, which might also allow explore the effects of non-teaching professionals in schools on future student educational outcomes such as entry, permanence, and graduation from higher education (Poynton and Lapan, 2017; O'Connor, 2018; Mulhern, 2020).

## Data Availability Statement

The raw data supporting the conclusions of this article will be made available by the authors, without undue reservation.

## Ethics Statement

The studies involving human participants were reviewed and approved by Pontificia Universidad Católica de Valparaíso. Written informed consent for participation was not required for this study in accordance with the national legislation and the institutional requirements.

## Author Contributions

All authors listed have made a substantial, direct and intellectual contribution to the work, and approved it for publication.

## Conflict of Interest

The authors declare that the research was conducted in the absence of any commercial or financial relationships that could be construed as a potential conflict of interest.
