# Supplementary material for: The Effect of School Psychologists and Social Workers on School Achievement and Failure: A National Multilevel Study in Chile
Source: Front Psychol. 2021 Feb 23;12:639089. doi: 10.3389/fpsyg.2021.639089 (PMC7940187; doi:10.3389/fpsyg.2021.639089)
Supplement: Supplementary file 1 [file Table_1.DOCX]

Supplementary Material

# Supplementary Table 1.

*Multilevel linear model predicting math and language score for 8^th^ grade and 10^th^ grade testing the contribution of the proportion of psychosocial professionals hired with indefinite and fixed-term contracts with individual and school-level predictors*

|  | **8th grade** | | **10th grade** | |
| --- | --- | --- | --- | --- |
|  | **Math score** | **Language Score** | **Math score** | **Language score** |
| **Variables** | **b (SE)** | **b (SE)** | **b (SE)** | **b (SE)** |
| ***Individual level*** |  |  |  |  |
| Female (Yes=1) | -6.86*** | 9.82*** | -8.08*** | 12.83*** |
|  | (0.23) | (0.26) | (0.34) | (0.32) |
| Age | -9.21*** | -8.54*** | -13.58*** | -10.33*** |
|  | (0.18) | (0.21) | (0.27) | (0.25) |
| Socioeconomic status | 7.64*** | 7.60*** | 7.93*** | 5.56*** |
|  | (0.19) | (0.21) | (0.25) | (0.24) |
| Indigenous ancestry | 0.58 | 1.21** | 1.37** | 0.42 |
|  | (0.34) | (0.38) | (0.50) | (0.47) |
| Attendance | 0.64*** | 0.18*** | 1.09*** | 0.40*** |
|  | (0.02) | (0.03) | (0.03) | (0.03) |
| School motivation | 5.82*** | 6.89*** | 10.95*** | 8.24*** |
|  | (0.17) | (0.20) | (0.24) | (0.22) |
| ***School level*** |  |  |  |  |
| Percentage of female students | 0.13*** | 0.08*** | 0.16*** | 0.14*** |
|  | (0.02) | (0.02) | (0.04) | (0.03) |
| *School SES (reference category: Low-SES school)* | | |  |  |
| Mid-low | 6.40*** | 4.10*** | 13.38*** | 6.94*** |
|  | (0.82) | (0.75) | (1.72) | (1.26) |
| Mid | 17.08*** | 11.75*** | 34.60*** | 17.06*** |
|  | (0.97) | (0.88) | (1.94) | (1.42) |
| Mid-high | 30.30*** | 19.16*** | 52.23*** | 23.80*** |
|  | (1.26) | (1.13) | (2.36) | (1.73) |
| High | 45.35*** | 24.46*** | 59.03*** | 29.20*** |
|  | (3.46) | (3.02) | (5.89) | (4.31) |
| Attendance (school average) | 1.13*** | 1.29*** | 1.72*** | 1.46*** |
|  | (0.08) | (0.07) | (0.13) | (0.10) |
| *School funding (reference category: public)* | |  |  |  |
| Subsidized private | 5.98*** | 1.44* | 3.08* | -2.03 |
|  | (0.70) | (0.62) | (1.51) | (1.10) |
| Private | 17.46*** | 6.83* | 15.41* | 0.67 |
|  | (3.67) | (3.19) | (6.12) | (4.46) |
| Rural school (Yes=1) | 0.34 | 1.51 | -6.92* | -2.45 |
|  | (0.87) | (0.80) | (2.88) | (2.15) |
| Percentage of psychosocial professionals with indefinite contract | 0.04 | 0.00 | -0.07 | 0.01 |
|  | (0.03) | (0.01) | (0.06) | (0.05) |
| Percentage of psychosocial professionals with fixed-term contract | 0.03 | 0.00 | -0.10 | -0.02 |
|  | (0.03) | (0.02) | (0.06) | (0.05) |
| Constant | 190.77*** | 202.06*** | 196.97*** | 221.37*** |
|  | (8.07) | (7.35) | (13.82) | (10.68) |
| Number of students | 123,574 | 123,574 | 85,636 | 85,636 |
| Number of schools | 4,536 | 4,536 | 1,824 | 1,824 |
| Log-likelihood | -627,995.1 | -643,569 | -450,384.7 | -444,171.5 |

Note: Unstandardized coefficients reported. Standard errors in parentheses. **p* < .05, ***p* < .01, ****p* <.001

# Supplementary Table 2.

*Multilevel linear model predicting math and language score for 8^th^ grade and 10^th^ grade testing the contribution of the proportion of psychosocial professionals hired with SEP and PIE funds with individual and school-level predictors*

|  | **8th grade** | | **10th grade** | |
| --- | --- | --- | --- | --- |
|  | **Math score** | **Language Score** | **Math score** | **Language score** |
| **Variables** | **b (SE)** | **b (SE)** | **b (SE)** | **b (SE)** |
| ***Individual level*** |  |  |  |  |
| Female (Yes=1) | -6.99*** | 9.68*** | -8.20*** | 12.60*** |
|  | (0.24) | (0.27) | (0.35) | (0.32) |
| Age | -9.38*** | -8.70*** | -13.75*** | -10.51*** |
|  | (0.19) | (0.21) | (0.28) | (0.26) |
| Socioeconomic status | 7.60*** | 7.59*** | 7.92*** | 5.46*** |
|  | (0.19) | (0.21) | (0.26) | (0.24) |
| Indigenous ancestry | 0.61 | 1.24** | 1.46** | 0.41 |
|  | (0.34) | (0.38) | (0.51) | (0.47) |
| Attendance | 0.64*** | 0.18*** | 1.08*** | 0.39*** |
|  | (0.02) | (0.03) | (0.03) | (0.03) |
| School motivation | 5.68*** | 6.74*** | 10.79*** | 7.94*** |
|  | (0.18) | (0.20) | (0.25) | (0.23) |
| ***School level*** |  |  |  |  |
| Percentage of female students | 0.13*** | 0.09*** | 0.17*** | 0.13*** |
|  | (0.02) | (0.02) | (0.04) | (0.03) |
| *School SES (reference category: Low-SES school)* | | | | |
| Mid-low | 6.30*** | 4.05*** | 12.81*** | 6.60*** |
|  | (0.82) | (0.75) | (1.74) | (1.27) |
| Mid | 16.84*** | 11.63*** | 34.01*** | 16.87*** |
|  | (0.97) | (0.89) | (1.97) | (1.44) |
| Mid-high | 30.46*** | 19.23*** | 51.79*** | 23.93*** |
|  | (1.27) | (1.15) | (2.42) | (1.77) |
| High | 46.86*** | 24.62*** | 57.33*** | 30.65*** |
|  | (3.68) | (3.21) | (6.40) | (4.65) |
| Attendance (school average) | 1.10*** | 1.27*** | 1.70*** | 1.44*** |
|  | (0.08) | (0.07) | (0.13) | (0.10) |
| *School funding (reference category: public)* | | | | |
| Subsidized private | 6.11*** | 1.48* | 3.17* | -1.70 |
|  | (0.70) | (0.62) | (1.51) | (1.09) |
| Rural school (Yes=1) | 0.84 | 1.81* | -6.27* | -1.95 |
|  | (0.88) | (0.81) | (2.93) | (2.18) |
| Percentage of psychosocial professionals hired with SEP funds | 0.02** | 0.01 | 0.01 | 0.02 |
|  | (0.01) | (0.01) | (0.02) | (0.01) |
| Percentage of psychosocial professionals hired with PIE funds | -0.02* | -0.01 | -0.06** | -0.03* |
|  | (0.01) | (0.01) | (0.02) | (0.02) |
| Constant | 199.08*** | 206.47*** | 195.05*** | 226.83*** |
|  | (7.71) | (7.42) | (12.60) | (9.77) |
| Number of students | 118,239 | 118,239 | 80,773 | 80,773 |
| Number of schools | 4,410 | 4,410 | 1,702 | 1,702 |
| Log-likelihood | -601,170.1 | -615,809.3 | -425,464.9 | -418,763.7 |

Note: Unstandardized coefficients reported. Standard errors in parentheses. **p* < .05, ***p* < .01, ****p* <.001
